# Effect of Atomic Layer Deposition of Ultra‐Thin Oxide on Reactivity and Durability of Perovskite Oxygen Electrodes

**DOI:** 10.1002/adma.202513655

**Published:** 2026-01-19

**Authors:** Jongsu Seo, SungHyun Jeon, Hyunseung Kim, San Kwak, DongHwan Oh, Bonjae Koo, Jinwook Kim, Jeong Hwan Kim, WooChul Jung

**Affiliations:** ^1^ Hydrogen Research Department Korea Institute of Energy Research (KIER) Daejeon Republic of Korea; ^2^ Research Institute of Advanced Materials Seoul National University Seoul Republic of Korea; ^3^ Department of Materials Science and Engineering Northwestern University Evanston Illinois USA; ^4^ Department of Materials Science and Engineering Seoul National University Seoul Republic of Korea; ^5^ Department of Chemical and Biomolecular Engineering (BK21 Four) Korea Advanced Institute of Science and Technology (KAIST) Daejeon Republic of Korea; ^6^ School of Chemistry and Energy Sungshin Women's University Seoul Republic of Korea; ^7^ Department of Materials Science and Engineering Hanbat National University Daejeon Republic of Korea; ^8^ School of Mechanical and Aerospace Engineering Nanyang Technological University (NTU) 50 Nanyang Drive Singapore

**Keywords:** atomic layer deposition, oxygen exchange kinetics, solid oxide fuel cell, Sr scavenger, strontium segregation

## Abstract

Conductive perovskite oxides (ABO_3_) are key oxygen electrode materials for energy conversion applications, but they suffer from irreversible performance degradation at elevated temperatures due to surface chemical instability. In this study, we investigate how the surface chemical environment and oxygen exchange kinetics of thin‐film La_0.6_Sr_0.4_CoO_3_ are affected by the atomic layer deposition of binary oxides—specifically HfO_2_ and Al_2_O_3_. We observe that both HfO_2_ and Al_2_O_3_ overcoats successfully maintain the rapid oxygen exchange rate on the electrode surface. Interestingly, however, their effects on the electrode surface chemistry differ significantly, as do the ideal coating thicknesses, as revealed by X‐ray photoelectron spectroscopy, secondary ion mass spectrometry, and transmission electron microscopy analyses. These findings suggest two distinct mechanisms for stabilizing the perovskite surface: the oxide overcoats (1) reduce oxygen vacancies that attract Sr ions to the surface and (2) act as a scavenger, consuming excess surface Sr, and suggest the design principle of a new strategy based on atomic layer deposition to improve perovskite surface durability.

## Graphical abstract

This table of contents illustrates that the mechanism for mitigating Sr segregation depends strongly on the chemistry of the coating layer. HfO_2_ electrostatically stabilizes the surface by lowering the surface oxygen vacancies, which serve as the driving force for Sr migration. On the other hand, Al_2_O_3_ and Fe_2_O_3_ react with segregated Sr to form Sr‐Al‐O & Sr‐Fe‐O intermediate compounds, effectively scavenging Sr at the surface. Together, these distinct behaviors underscore that the optimal mitigation of Sr segregation requires carefully tuning coating parameters and operation conditions in accordance with the chemical properties and reactivities of the coating materials.

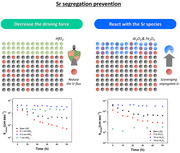

## 1 Introduction

Solid oxide fuel cells (SOFCs) have gained significant attention as a promising next‐ generation power source due to their high energy conversion efficiency and eco‐friendliness [1, 2]. To achieve high‐performance SOFCs, it is essential to develop oxygen electrodes with both high activity toward oxygen reduction reaction‐ the rate‐limiting step‐and long‐term durability at elevated temperatures [3, 4]. Mixed ionic and electronic conducting perovskite oxides (i.e., ABO_3_), such as (La,Sr)MnO_3_, (La,Sr)(Co,Fe)O_3_, (Ba,Sr)(Co,Fe)O_3_, are widely used as promising oxygen electrode materials because of their excellent transport properties and favorable surface oxygen exchange kinetics [4, 5, 6, 7]. However, the surface chemical instability of such electrodes at elevated temperatures remains a critical challenge for practical applications. Taking Sr‐containing perovskite oxide as an example, the formation of SrO‐like insulating secondary phase on the surface and the presence of nonstoichiometric subsurface regions has been suggested as key causes of irreversible performance degradation by impeding electron transfer and/or oxygen exchange kinetics [8, 9, 10, 11]. Therefore, a variety of studies have been conducted, focusing on how to reduce surface chemical heterogeneity to improve the durability of these materials.

To mitigate surface chemical heterogeneity, protective coatings have been proposed as an effective solution to stabilize the electrode surface and thereby maintain stable oxygen exchange kinetics over time at elevated temperatures. For example, Yildiz et al. suggested that surface modification with binary oxides containing less reducible cations, such as Al_2_O_3_, ZrO_2_, and HfO_2_, can effectively stabilize the La_0.8_Sr_0.2_CoO_3_ surface by suppressing surface oxygen vacancies (V_o_^••^) that electrostatically attract Sr^'^_La_. Compared to pristine La_0.8_Sr_0.2_CoO_3_, the HfO_2_‐modified surface exhibited a 30 fold enhancement in oxygen exchange rate after 54 h at 530°C [12]. Based on these findings, they proposed that the oxygen vacancy formation enthalpy of coating materials serves as a key descriptor for selecting materials that can inhibit Sr segregation. In the same vein, Huang et al. reported that a 0.8 nm layer of ZrO_2_ coating on La_0.6_Sr_0.4_CoO_3_ cathode decreases surface deformation caused by Sr segregation, thereby enhancing the electrode activity without significant degradation compared to the pristine electrode [13].

However, despite promising reports on enhanced oxygen exchange kinetics, some studies have demonstrated divergent or even adverse effects of oxide coating on electrode performance—sometimes using the very same materials. For example, Hwang et al. reported that the deposition of Al_2_O_3_ on LSC hinders the adsorption/dissociation of oxygen molecules or charge transfer at the interface by blocking active sites, thereby reducing electrode activity [14]. Similarly, Gorte et al. reported that even with a very small amount of ZrO_2_ coating (i.e., 1 wt. % or less), the polarization resistance of the La_0.8_Sr_0.2_FeO_3_ electrode increased because of the effect of blocking the oxygen adsorption site on the surface [15, 16]. Moreover, conflicting interpretations have been reported regarding how ZrO_2_ coatings mitigate Sr segregation. One study points to space charge effects as the primary mechanism, whereas another highlights the formation of an SrO‐ZrO_2_ intermediate phase as a scavenging mechanism [17, 18].

In sum, successful electrode performance and durability improvement have been reported using surface modification, but there are still many controversies about the impact of surface coating on electrode activity. This is partly due to the chemical and morphological complexity of the perovskite oxide surface and the challenge of uniformly controlling coating layers. As a result, several fundamental questions remain unanswered: “Can an oxide overcoat truly suppress surface reactivity degradation of perovskite electrodes?” “If so, which coating materials are most effective, and what is the ideal coating thickness?” Finally, “what mechanisms underlie the improved durability imparted by oxide coatings?”

To address these issues, we employed a model thin‐film electrode platform that combines pulsed layer deposition (PLD) and atomic layer deposition (ALD) [19, 20, 21]. Flat and dense polycrystalline LSC thin films were deposited on Al_2_O_3_ substrates by PLD, followed by con‐ formal oxide coatings through ALD [22, 23, 24, 25]. The surface oxygen exchange rates were measured depending on two types of coating material (Al_2_O_3_ or HfO_2_) and overcoat thickness using the electrical conductivity relaxation (ECR) method. Simultaneously, the physical and chemical attributes of each coated sample were characterized using a range of analytical tools, including scanning electron microscopy (SEM), X‐ray photoelectron spectroscopy (XPS), time‐of‐flight secondary ion mass spectrometry (ToF‐SIMS), and transmission electron microscopy (TEM). We observed that both Al_2_O_3_ and HfO_2_ coatings successfully sustain the fast oxygen exchange kinetics of LSC without degradation; however, their interfacial chemistries differ significantly. Although HfO_2_ suppresses the surface deformation of LSC caused by Sr segregation, as reported in previous studies, Al_2_O_3_ does not mitigate Sr enrichment. Instead, new compounds resulting from the reaction between Al_2_O_3_ and segregated Sr were observed on the surface of Al_2_O_3_‐coated LSC. These results indicate that the reactivity of coating materials with Sr, which enables scavenging excess surface Sr, can serve as a critical design criterion for selecting effective coatings. Based on this insight, we further propose Fe_2_O_3_ as a promising Sr‐scavenging overcoat that achieves active and stable oxygen exchange kinetics at 600°C.

## 2 Experimental Details

### 2.1 Sample Preparation

The LSC powder was synthesized via a conventional sol‐gel method to fabricate a dense ceramic target for pulsed laser deposition (PLD). Stoichiometric amounts of lanthanum nitrate, strontium nitrate, and cobalt nitrate were dissolved in deionized water at a molar ratio of 0.6:0.4:1.0. Citric acid and ethylenediaminetetraacetic acid (EDTA) were added as chelating agents in a metal:citric acid:EDTA molar ratio of 2:3:1. The pH of the solution was adjusted to 9 using ammonium hydroxide (NH_4_OH), ensuring complete dissolution of the metal complexes. The resulting solution was stirred at 170°C overnight for gelation, followed by combustion at 450°C for 3 h using a heating mantle. The obtained powder was calcined at 900°C for 8 h in a box furnace, uniaxially pressed, and sintered at 1150°C for 5 h to prepare the PLD target.

LSC thin films with a thickness of approximately 400 nm were deposited on single‐crystal Al_2_O_3_ (sapphire) substrates by pulsed laser deposition using a KrF excimer laser (λ = 248 nm, Lambda Physik COMPex 205) operating at 300 mJ and 10 Hz. The substrate temperature was maintained at 700°C during deposition, and the working pressure of the chamber, filled with high‐purity oxygen gas (99.999 %), was controlled at 10 mTorr. An Al_2_O_3_ single‐crystal substrates were used because they provide an insulating and flat surface. Unlike SrTiO_3_ (STO) substrates, which are often employed to induce epitaxial growth due to their lattice matching [26, 27] Al_2_O_3_ substrate allows polycrystalline growth of LSC thin films without preferred orientation, thus offering conditions closer to realistic electrodes [28].

The chemical etching was conducted on the LSC thin film in a 0.1 m HCl aqueous solution for 15 s, followed by rinsing with deionized water for over 30 s and subsequent drying.

An Al_2_O_3_ coating layer was deposited onto the LSC thin film via atomic layer deposition (ALD) using trimethylaluminum (TMA) as the Al precursor and diluted water as the oxidant at a substrate temperature of 230°C. Each ALD cycle consisted of a TMA pulse (0.5 s), Ar purge (5 s), water pulse (1 s), and a final Ar purge (15 s).

HfO_2_ was deposited using tetrakis(ethylmethylamino)hafnium (TEMAHf) as the Hf precursor and water (H_2_O) as the oxidant at substrate temperatures of 250°C. Each ALD cycle consisted of a TEMAHf pulse (1.5 s), Ar purge (30 s), water pulse (1 s for standard conditions), and a final Ar purge (30 s).

PLD was employed to deposit an Fe_2_O_3_ coating layer on the LSC thin film. The Fe_2_O_3_ target was first fabricated using uniaxial pressing, followed by sintering at 1200°C. The deposition was carried out at room temperature under a working pressure of 10 mTorr with O_2_. The deposition time was 30 s corresponded to a thickness of 1 nm.

To mimic the actual electrode structure, we also fabricated an SOFC half‐cell with porous La_0.6_Sr_0.4_CoO_3‐d_ (LSC) electrodes. LSC ink slurry was prepared by mixing an ink vehicle and LSC powder in a 1:1 weight ratio along with a small amount of ethanol, and the resulting ink was screen‐printed onto the both sides of 8 mol %‐Y_2_O_3_‐stabilized ZrO_2_ (YSZ) electrolytes with PLD‐deposited Gd_0.1_Ce_0.9_O_2‐d_ buffer layers. The half‐cells were then sintered at 950°C for 1 h in air. The Fe_2_O_3_ was incorporated on both sides of porous LSC electrodes. To better reflect a practical electrode fabrication route, the infiltration process was selected for Fe_2_O_3_ coating rather than thin‐film deposition. A 10 mM Fe(NO_3_)_3_∙9H_2_O solution (10 µL per electrode side) was infiltrated, followed by calcination at 650°C. The electrochemical impedance spectroscopy stability of pristine LSC and Fe_2_O_3_‐coated LSC porous half‐cell were compared to determine whether the Fe_2_O_3_ coating is effective in an actual porous electrode configuration.

### 2.2 Physical Characterization

X‐ray diffraction (XRD, D8‐Advance (XRD, a1 system)) analysis of the as‐prepared and heat‐treated powder was conducted. The morphology of as‐prepared and heat‐treated LSC with and without oxide coating was examined by scanning electron microscopy (SEM, Hitachi S‐4800). A high‐angle annular dark‐field scanning transmission electron microscopy (HAADF‐STEM, Talos F200X) at 200 kV and corresponding EDS mapping were employed to confirm the changes in surface morphology and composition.

Depth profiling of the LSC thin film surface was performed using time‐of‐flight secondary ion mass spectrometry (ToF‐SIMS5, ION‐TOF GmbH, Germany). A 30 keV Bi^−^ ion beam was used to scan a 100 × 100 µm^2^ area for secondary ion generation, while a 2 keV Cs^+^ ion beam was employed to sputter a 300 × 300 µm^2^ area for depth profiling. The positive secondary ion mode was used to detect La^+^, Sr^+^, SrO^+^, AlO^+^, and SrAlO^+^ signals.

### 2.3 Electrical Conductivity Relaxation (ECR) Analysis

To evaluate the electrical conductivity of the LSC thin films, two platinum electrodes (thickness: ∼200 nm; spacing: 2 mm) were deposited on the film surface by DC magnetron sputtering through a metal shadow mask. The sputtering was conducted at a DC power of 10 W under 10 mTorr of Ar atmosphere with a flow rate of 30 sccm, yielding a deposition rate of approximately 60 nm·min^−1^. Prior to the conductivity measurements, surface‐segregated Sr species formed during the PLD process were removed by immersing the samples in a 0.1 m HCl solution for 15 s.

To evaluate the electrical conductivity of the LSC thin films, two platinum electrodes (thickness: ∼200 nm; spacing: 2 mm) were deposited on the film surface by DC magnetron sputtering through a metal shadow mask. The sputtering was conducted at a DC power of 10 W under 10 mTorr of Ar atmosphere with a flow rate of 30 sccm, yielding a deposition rate of approximately 60 nm·min^−1^. Prior to the conductivity measurements, surface‐segregated Sr species formed during the PLD process were removed by immersing the samples in a 0.1 m HCl solution for 15 s.

The measurements were carried out in a tube furnace equipped with a K‐type thermocouple at 530°C under a controlled gas atmosphere consisting of O_2_ and Ar. The oxygen partial pressure (*p*O_2_) was varied between 0.21 and 1 atm with the total flow rate maintained at 100 sccm using mass flow controllers. After equilibrating the sample at each condition, a sudden change in *p*O_2_ was introduced via a four‐way valve, keeping the temperature constant. The in‐plane electrical conductivity, which reflects changes in the oxygen stoichiometry of the film, was monitored by applying a constant DC current (700 µA) and recording the resulting voltage at 0.1 s intervals using chronopotentiometry (CP, VSP‐300, BioLogic) until the sample reached a new equilibrium.

The effective surface exchange coefficient (k) was then obtained by fitting the conductivity relaxation profiles to a first‐order surface exchange model:

$$\frac{\sigma \left(t\right)-\sigma \left(0\right)}{\sigma \left(\infty \right)-\sigma \left(0\right)}=1-\exp\left(-\frac{k}{\alpha }t\right)$$

where *σ*(t) is the conductivity at time *t*, *σ*(0) and *σ*(∞) are the initial and equilibrium conductivities, respectively, and α is the film thickness.

## 3 Results and Discussion

First, we obtained a well‐defined LSC thin film without deformation due to Sr segregation to accurately evaluate the effect of oxide coating on the surface oxygen exchange kinetics of LSC. As shown in Figure S1a, the LSC thin film fabricated using PLD is inevitably chemically heterogeneous at the surface owing to Sr segregation, because the deposition process is performed at high temperatures. Thus, we carefully conducted chemical etching [28], achieving a clean and flat polycrystalline LSC thin film (Figure S1b). Figure S2 also supports that the surface Sr‐segregation layer was removed after chemical etching.

**FIGURE 1:**
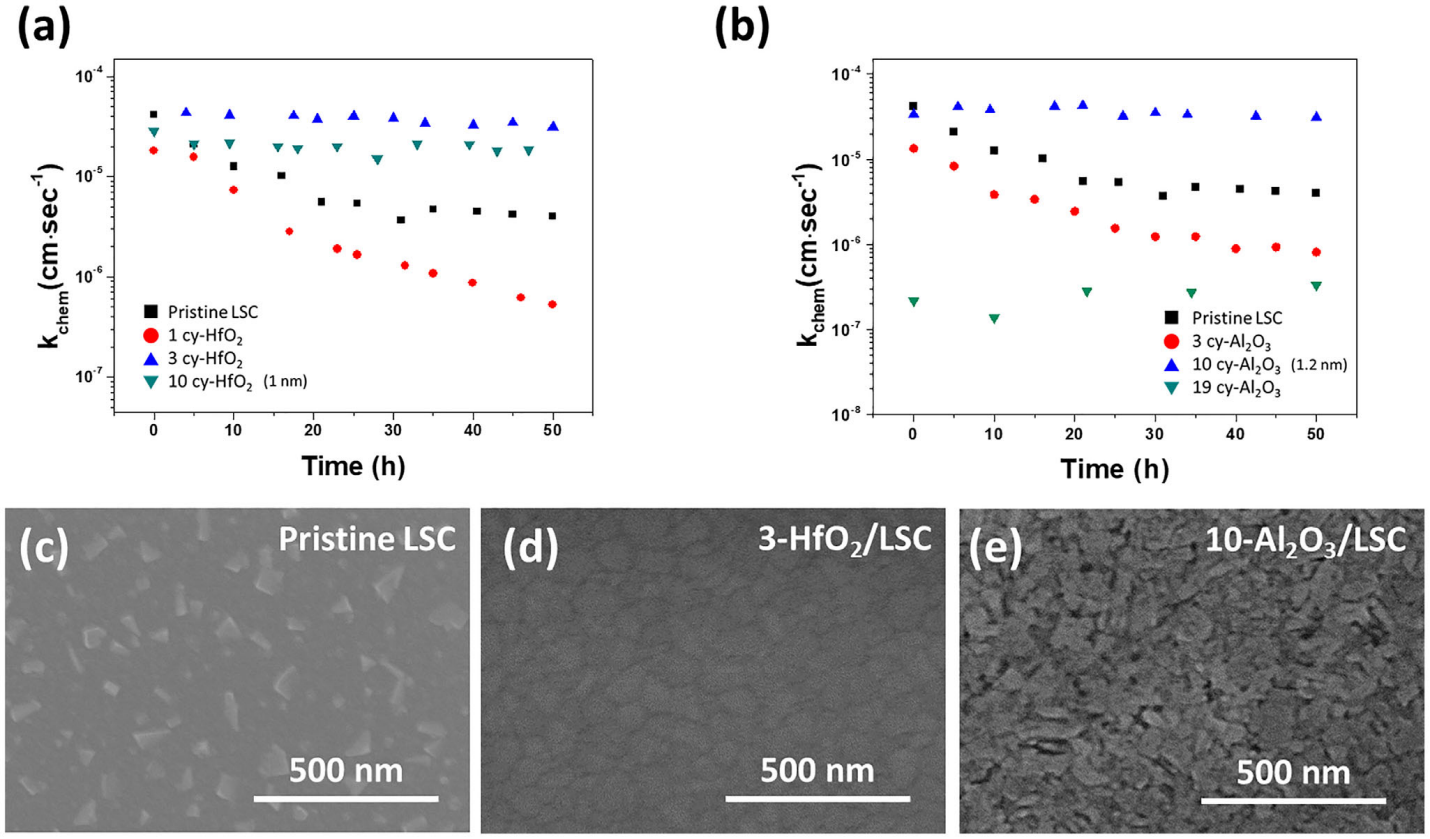
Surface oxygen exchange kinetics and stability on LSC dense thin film cathode and its surface morphology. Oxygen exchange coefficient depending on the thickness of (a) HfO_2_ and (b) Al_2_O_3_ coating using atomic layer deposition at 530°C for 50 h. The SEM images of (c) pristine LSC, (d) 3 cy of HfO_2_‐coated LSC, and (e) 10 cy of Al_2_O_3_‐coated LSC after measurement.

Next, we measured the surface oxygen exchange coefficient with and without surface coating using the obtained clean LSC film by ECR measurement. Hereafter, we refer to the LSC film with an HfO_2_ or Al_2_O_3_ overcoat as the *x*‐HfO_2_/LSC or *x*‐Al_2_O_3_/LSC (with *x* indicating the number of ALD cycles). Detailed information on the ALD process is provided in the supporting materials. As shown in Figure 1a, pristine LSC showed the value of the oxygen exchange coefficient, 4.2 × 10^−5^ cm^−1^ right after elevating the temperature to 530°C and a continuous decrease over time, which is commonly attributed to degradation caused by surface Sr segregation.

To evaluate the potential of oxide overcoats in suppressing such degradation, we prepared HfO_2_‐coated LSC thin films using 1, 3, and 10 ALD cycles, with 10 cycles corresponding to approximately 1 nm in thickness (Figure S3). The 1‐HfO_2_/LSC sample exhibited a similar degradation trend to that of the pristine LSC, likely due to insufficient coating coverage. In contrast, the 3‐HfO_2_/LSC sample demonstrated both high oxygen exchange kinetics and stable performance over time. Likewise, when the coating cycle increased further (10‐HfO_2_/LSC), the sample also demonstrated great stability over time, but exhibited a slight reduction in oxygen exchange kinetics, which is likely attributed to the excessive thickness of the overcoat limiting surface reactivity. Thus, the effective thickness is 3 cycles (much less than 1 nm) for HfO_2_. The improved oxygen exchange stability of LSC with HfO_2_ is as reported in the literature, due to the incorporation of less reducible Hf cation near the B‐sites, which strengthens the metal‐oxygen bonds, increasing the oxygen vacancy formation enthalpy, thereby reducing oxygen vacancies in LSC. [12, 29]

A similar trend was observed for Al_2_O_3_‐coated LSC, where increasing the number of ALD cycles led to enhanced stability. However, the critical thickness for effective stabilization was different. The 3‐Al_2_O_3_/LSC sample showed degradation behavior comparable to that of the pristine LSC. Stabilization became evident at 10 ALD cycles (∼1.2 nm), and the 19‐Al_2_O_3_/LSC sample demonstrated further stability, albeit with slightly suppressed oxygen exchange activity due to overpassivation. Considering that HfO_2_ and Al_2_O_3_ exhibit nearly identical ALD growth rates, the difference in their critical thicknesses required for stabilization is particularly noteworthy.

The SEM images after ECR measurement further support these results (Figure 1c–e). One of the major visual indicators of Sr segregation is the formation of surface SrO‐like insulating phases, often exhibiting angular morphologies. As shown in Figure 1c, the uncoated LSC thin film displays numerous triangular surface precipitates after 50 h of testing, indicating significant degradation [30, 31]. In contrast, the 3‐HfO_2_/LSC sample, shown in Figure 1d, maintains a clean surface without any detectable secondary phases, even after undergoing the same ECR test. Similarly, the 10‐Al_2_O_3_/LSC sample also shows no visible angular precipitates (Figure 1e), although a slight change in surface roughness was observed, which will be discussed in a later section. These morphological findings are in good agreement with the ECR stability results.

One of the primary mechanisms of surface segregation of aliovalent dopants in perovskite oxides has been proposed to involve the electrostatic attraction of the negatively charged dopants (e.g, Sr^'^_La_) by the positively charged oxygen vacancies (Vo^••^) [12]. Therefore, using coatings of less reducible oxides, such as HfO_2_, Al_2_O_3_, and ZrO_2_, to control the concentration of oxygen vacancies at the surface of perovskite oxides has been suggested as an effective strategy to improve the chemical stability of oxygen exchange kinetics. Since oxygen vacancies also function as active sites in the oxygen exchange reaction [32], there exists a trade‐off between reducing their concentration to improve surface stability and retaining enough vacancies to maintain sufficient oxygen exchange activity. Accordingly, HfO_2_, which has a moderate oxygen vacancy formation enthalpy, is considered the most effective coating material among the oxides studied [12]. However, in this study, we found that both HfO_2_ and Al_2_O_3_ can achieve comparable oxygen exchange kinetics when the number of ALD cycles is carefully controlled, with the appropriate cycle numbers identified as 3 for HfO_2_ and 10 for Al_2_O_3_, respectively. It is worth noting that the oxygen vacancy formation enthalpy of Al_2_O_3_ is significantly higher than that of HfO_2_. If the sole role of the coating layer were to control surface oxygen vacancy concentration, as previously assumed, then Al_2_O_3_ would be expected to require a thinner coating than HfO_2_, due to its stronger oxygen bonding strength. However, our result that Al_2_O_3_ requires a much thicker coating than HfO_2_ to achieve stabilization suggests that factors beyond oxygen bonding energy may also play an important role in improving oxygen exchange kinetics.

**FIGURE 2:**
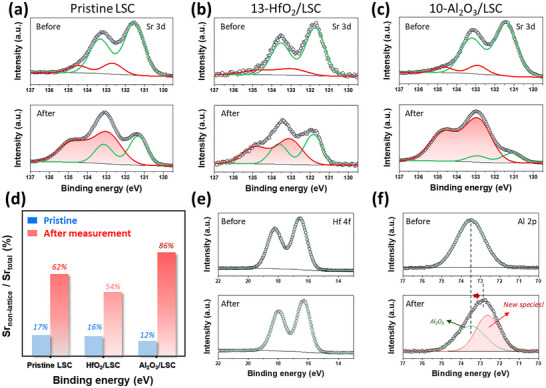
X‐ray photoelectron spectroscopy of LSC with and without ALD coating. The Sr 3d core‐level peak spectrum recorded from (a) pristine LSC, (b) 13‐HfO_2_/LSC, and (c) 10‐Al_2_O_3_/LSC before and after measurement. (d) The summarized graph of the Sr_non‐lattice_/Sr_total_ ratio before and after measurement. The (d) Hf 4f and (e) Al 2p core‐level peak spectrum recorded from 13‐HfO_2_ and 10‐Al_2_O_3_ before and after measurement.

To investigate the detailed mechanism behind the improvement in stability for oxygen exchange kinetics, we conducted XPS analysis on pristine LSC, 13‐HfO_2_/LSC, and 10‐Al_2_O_3_/LSC samples before and after operation at 530°C for 50 h. Since XPS is a surface‐sensitive technique, samples with similar coating thicknesses (∼1.2 nm) were selected to minimize spectral distortion between samples [33, 34]. Figure 2 displays Sr 3d spectra of samples before and after operation. As shown in Figure 2, the Sr 3d spectra were well‐fitted using two sets of spin‐orbit doublets with an energy separation of ∼ 1.8 eV and an area ratio of 1.5. The Sr 3d doublet at lower binding energy, fitted with a green line, corresponds to Sr in the bulk lattice, Sr_lattice_, whereas the other doublet with higher binding energy, fitted with a red line, originates from the surface Sr species, Sr_non‐lattice_ [10]. In general, Sr_non‐lattice_ peaks are known to indicate insulating Sr‐rich particles or layers such as SrO, Sr(OH)_2_, or Sr(CO)_3_ that are on the LSC surface owing to Sr segregation. The chemical environment before and after measurement was evaluated by the comparison of the area ratio of “Sr_non‐lattice_/Sr_total_” [10, 33]. As summarized in Figure 2d, pristine LSC, 13‐HfO_2_/LSC, and 10‐Al_2_O_3_/LSC films showed distinct characteristics in their Sr 3d spectra during heat treatment. For the pristine LSC (Figure 2a), the Sr_non‐lattice_ ratio increased from 17 % to 62 % after heat treatment, indicating significant formation of surface Sr species. In contrast, the 13‐HfO_2_/LSC sample showed a much smaller increase in the Sr_non‐lattice_ fraction under the same treatment conditions (Figure 2b). This indicates that the HfO_2_ coating effectively suppresses Sr enrichment at the surfaces, leading to achieving the fast oxygen exchange kinetics with high durability [12]. On the other hand, the 10‐Al_2_O_3_/LSC exhibited a considerably greater increase in the Sr_non‐lattice_ peak after heat treatment, which at first glance may suggest limited suppression of Sr segregation. However, considering that both HfO_2_‐ and Al_2_O_3_‐coated LSC exhibited similar oxygen exchange stabilities over time, this difference in surface chemical nature implies that the mechanisms behind the performance improvement may differ between the two coatings. To further investigate the origin of this difference, we also examined changes in the Hf 4d and Al 2p spectra before and after heat treatment. For 13‐HfO_2_/LSC, there was no remarkable change in the Hf 4f spectrum before and after measurement; however, the Al 2p peak in 10‐Al_2_O_3_/LSC exhibited a significant shift and appeared as multiple species after measurement. These observations indicate that even though 10‐Al_2_O_3_/LSC shows comparable oxygen exchange kinetics and durability compared to 3‐HfO_2_/LSC, Sr segregation on the surface of 10‐Al_2_O_3_/LSC was not prevented effectively, and a notable chemical change occurred within the Al_2_O_3_ layer.

**FIGURE 3:**
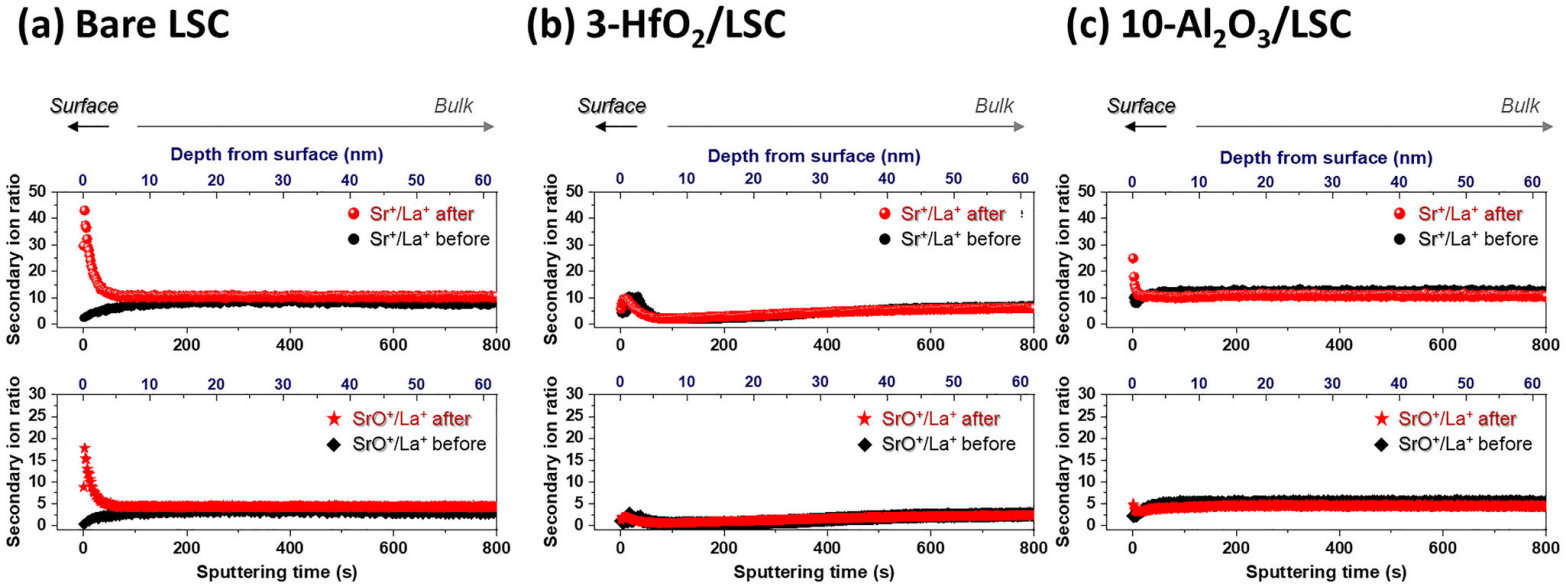
Depth profiling of LSC films depending on surface coating. Positive secondary ion signal ratio of (a) pristine LSC, (b) 3‐HfO_2_/LSC, and (c) 10‐Al_2_O_3_/LSC. The graphs show the changes in Sr^+^/La^+^ and SrO^+^/La^+^ ratios before and after operation, reflecting the degree of Sr segregation toward the surface.

To investigate the chemical environment near the surface region in more detail, depth profiling of cation composition was investigated by ToF‐SIMS, which is a highly surface‐sensitive technique capable of probing the sub‐nanometer‐scale region, both before and after the measurement [27, 35]. Since our primary interest lies in the behavior of Sr species, La—whose concentration is known to remain relatively stable—was used as a reference element. Therefore, the secondary ion ratio of Sr species to La was analyzed to assess changes in the relative surface composition. Figure 3 displays the secondary ion ratios of Sr^+^/La^+^ and SrO^+^/La^+^ before and after measurement for comparison of relative chemical composition with and without ALD coating. Due to the characteristics of the analysis equipment, the values of the ratios cannot be used as quantitative information, but alterations between the ratios for different films are of importance and indicate true differences in the cation composition. For bare LSC, the ratio of both Sr^+^/La^+^ and SrO^+^/La^+^ increased toward the surface after measurements (Figure 3a). This indicates that Sr species became abundant at the surface, forming Sr‐rich phases such as SrO or Sr(OH)_2_. In contrast, as shown in Figure 3b, the ratio of Sr^+^/La^+^ and SrO^+^/La^+^ was almost identical before and after measurement at the 3‐HfO_2_/LSC. In line with previous literature, we further confirm that the HfO_2_ coating effectively suppresses the Sr enrichment at the surface, thereby achieving fast oxygen exchange kinetics with high durability in ECR [12]. For the case of Al_2_O_3_‐coated LSC, the ratio of Sr^+^/La^+^ increased toward the surface after measurement (Figure 3c). However, compared to the dramatic increase in the ratio of the Sr_non‐lattice_ peak shown in previous XPS results (Figure 2c), the rate of increase toward the surface of Sr^+^/La^+^ at the 10‐Al_2_O_3_/LSC was lower than that of the bare LSC. This is discussed later with the cross‐sectional TEM results. In addition, while the ratio of Sr^+^/La^+^ at the surface of 10‐Al_2_O_3_/LSC increased noticeably, the ratio of SrO^+^/La^+^ remained nearly unchanged before and after measurement. Supporting this observation, Figure S4 shows that the ratio of SrAlO^+^/La^+^ on the 10‐Al_2_O_3/_LSC surface increased after measurement, while the ratio of AlO^+^/La^+^ decreased. Taken together, these findings suggest that Al_2_O_3_ cannot effectively depress Sr enrichment at the surface. Instead, Sr at the Al_2_O_3‐_coated LSC surface exists in a different chemical phase, likely formed through its reaction with the Al_2_O_3_ overlayer, in contrast to the pristine LSC surface. This possibility is also supported by the phase diagram reported between SrO and the coated oxide. Unlike HfO_2_, which is reported to react with SrO only at elevated temperatures above 900°C [36, 37]. Al_2_O_3_ can readily form various intermediate phases with SrO at even relatively low temperatures around 530°C, such as SrAl_2_O_4_, Sr_3_Al_2_O_6_, Sr_4_Al_14_O_25_, and Sr_12_Al_14_O_33_ [38, 39]. X‐ray diffraction (XRD) analysis of a SrO‐Al_2_O_3_ mixed powder subjected to heat treatment at 530°C provides additional evidence for the formation of intermediate phases, including SrAl_4_O_7_, Sr_4_Al_14_O_25_, and Sr_4_Al_2_O_7_ (Figure S5) [40]. The XRD results clearly indicate that such reactions do not occur in the SrO‐HfO_2_ system, whereas they readily proceed even at lower temperatures in the SrO + Al_2_O_3_ system, highlighting the much higher reactivity and scavenging ability of Al_2_O_3_ toward SrO. Therefore, taken together, we can conclude that Sr enrichment at the surface of Al_2_O_3_‐coated LSC is slightly decreased compared to the pristine LSC, but quite a lot of Sr segregation is still observed. Importantly, however, the different point is that the segregated Sr at the Al_2_O_3_‐coated LSC surface does not exist in the same phase as SrO or Sr(OH)_2_, as observed on the surface of pristine LSC, but instead forms a distinct compound through which the Al_2_O_3_ overcoat. Such a difference is also evident from the BET observation of surface roughness changes in LSC/Al_2_O_3_ before and after heat treatment (Figure S6 and Table S1).

**FIGURE 4:**
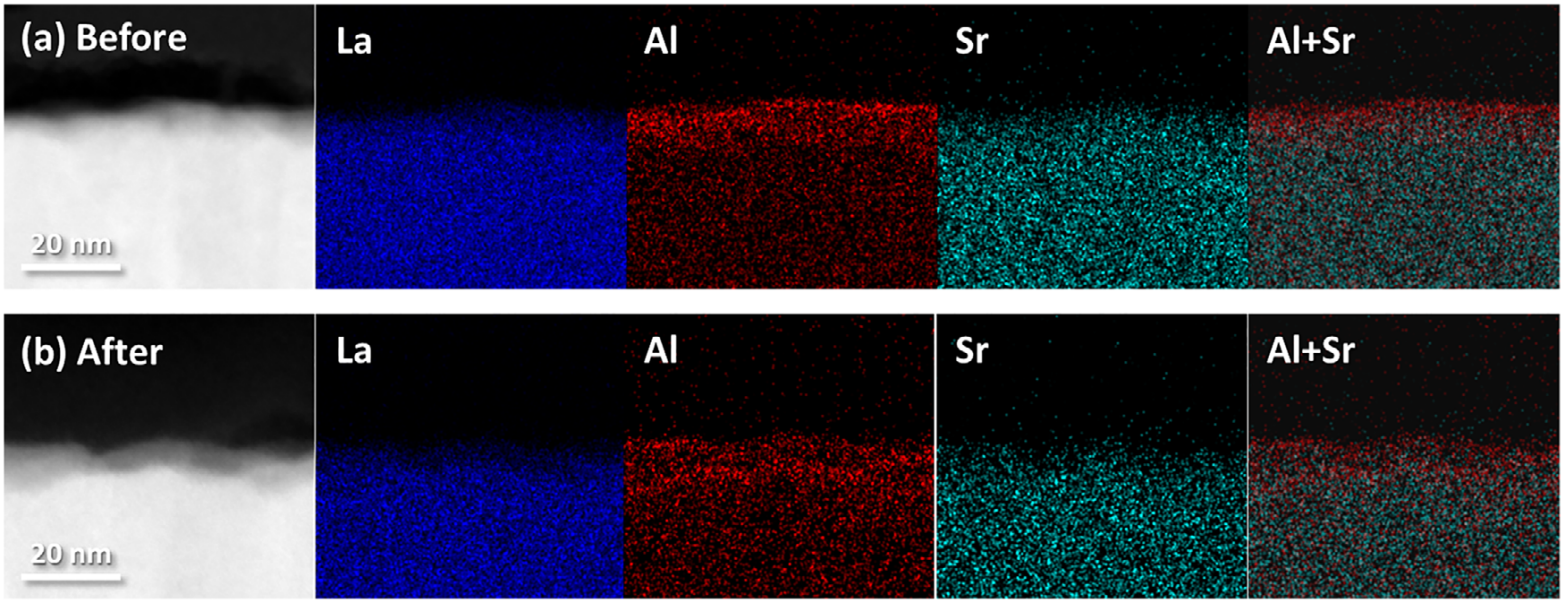
Cross TEM images of 20‐Al_2_O_3_/LSC films before and after heat treatment. High‐resolution TEM images of Al_2_O_3_‐coated LSC (a) before and (b) after heat treatment with their corresponding mapping profile.

To gain further insight into the interaction between Sr and Al_2_O_3_, we conducted TEM analysis to determine the distribution of Al, La, and Sr elements in the 20‐Al_2_O_3_/LSC. For ease of observation, a thick coating was selected for analysis. The thickness of Al_2_O_3_ is similar to that calculated using the growth rate obtained on the Si wafer. As shown in Figure 4, Al was spread evenly over the top surface of the LSC, while La and Sr were only located inside the LSC lattice before the heat treatment. However, after heat‐treatment at 530°C for 50 h, the Al_2_O_3_ coating became slightly thicker, and Sr was found to be diffused from the LSC lattice into the Al_2_O_3_ layer, while La was still only located inside the LSC lattice, as confirmed by multiple TEM analyses (Figures S7 and S8). This is the evidence for the possible chemical reaction between segregated Sr and the Al_2_O_3_ layer. A similar phenomenon has been observed for CeO_2_, where even trace amounts of Si impurities significantly reduce surface activity by blocking active sites. To solve this problem, Bishop et al. reported that La coating using PLD effectively improved the surface activity by scavenging Si impurities [41]. They suggested that the formation of lanthanum silicate, accompanied by cracking, enhanced surface activity to penetrate the oxygen gases into the Pr‐doped CeO_2_. More recently, Nicholas et al. reported that an overcoat of ZrO_2_ on an LSCF‐GDC nanocomposite cathode removed the deleterious surface Sr via the formation of SrZrO_3_ [18]. In a similar manner, we hypothesize that the mechanism for improving electrode durability and activity using Al_2_O_3_ coating originates from a volumetric change in the reaction between segregated Sr and Al_2_O_3_ involving cracking, which allows gas to penetrate the LSC film. It is also helpful to understand the dramatic increment of the Sr_non‐lattice_ peak in the XPS results compared with the ToF‐SIMS results. Since XPS is a surface‐sensitive technique, the ratio of the measured Sr_lattice_ inside the LSC decreases when the coating layer becomes thicker. Thus, as the thickness of the coating layer increases owing to the reaction between Sr and Al_2_O_3_ inside the coating layer after measurement, a significant increase in the Sr_non‐lattice_ peak, as shown in the previous XPS results (Figure 2c), is observed. This interpretation is further supported by the increased surface roughness observed in the SEM analysis of the LSC/Al_2_O_3_ sample after heat treatment (Figure 1e).

**FIGURE 5:**
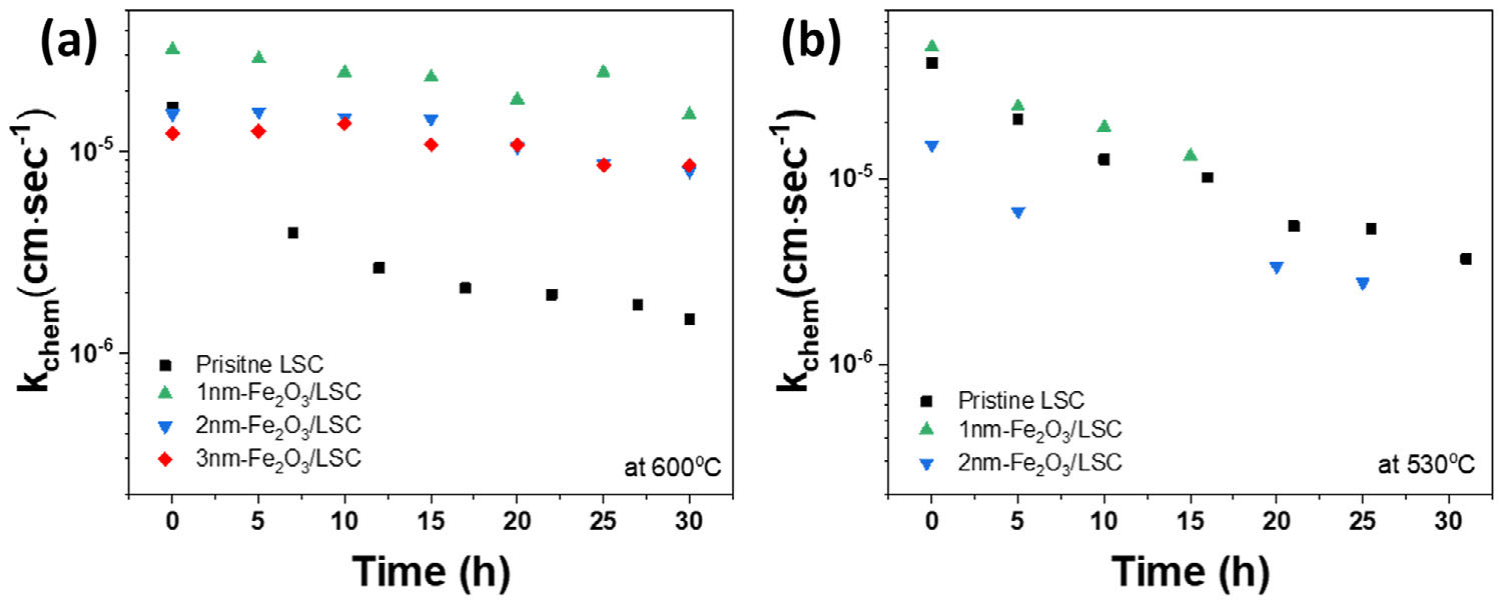
Time evolution of surface oxygen exchange kinetics on LSC dense thin film cathode at (a) 600°C and (b) 530°C. The oxygen exchange coefficient depends on the thickness of Fe_2_O_3_. Fe_2_O_3_ was deposited on LSC film via PLD at room temperature.

To verify the validity of the above findings and interpretations, Fe_2_O_3_ was employed as a new coating material to stabilize the oxygen exchange kinetics of LSC, given its distinct reactivity with SrO at different temperature ranges. According to the phase diagram, Fe_2_O_3_ can also form intermediate phases with various compositions [42, 43]. To verify the ability of proposed materials, we deposited Fe_2_O_3_ thin film with varying thickness via PLD on LSC films and measured its oxygen exchange kinetics through ECR. We found that Fe_2_O_3_ coating enhances both the oxygen exchange kinetics and the stability of the LSC film at 600°C (Figure 5a; Figure S9). Interestingly, however, such improvement in durability was not observed at 530°C, despite using the same coating material and thickness (Figure 5b). XRD analysis of SrO‐Fe_2_O_3_ mixed powders heat‐treated at 530°C and above 600°C, respectively, reveals a clear difference in phase evolution, with the possible formation of Sr‐Fe‐O intermediate phases, such as Sr_2_Fe_2_O_5_, observed only at temperatures above 600°C, indicating a temperature‐dependent stabilization behavior (Figure S10) [44, 45].

**FIGURE 6:**
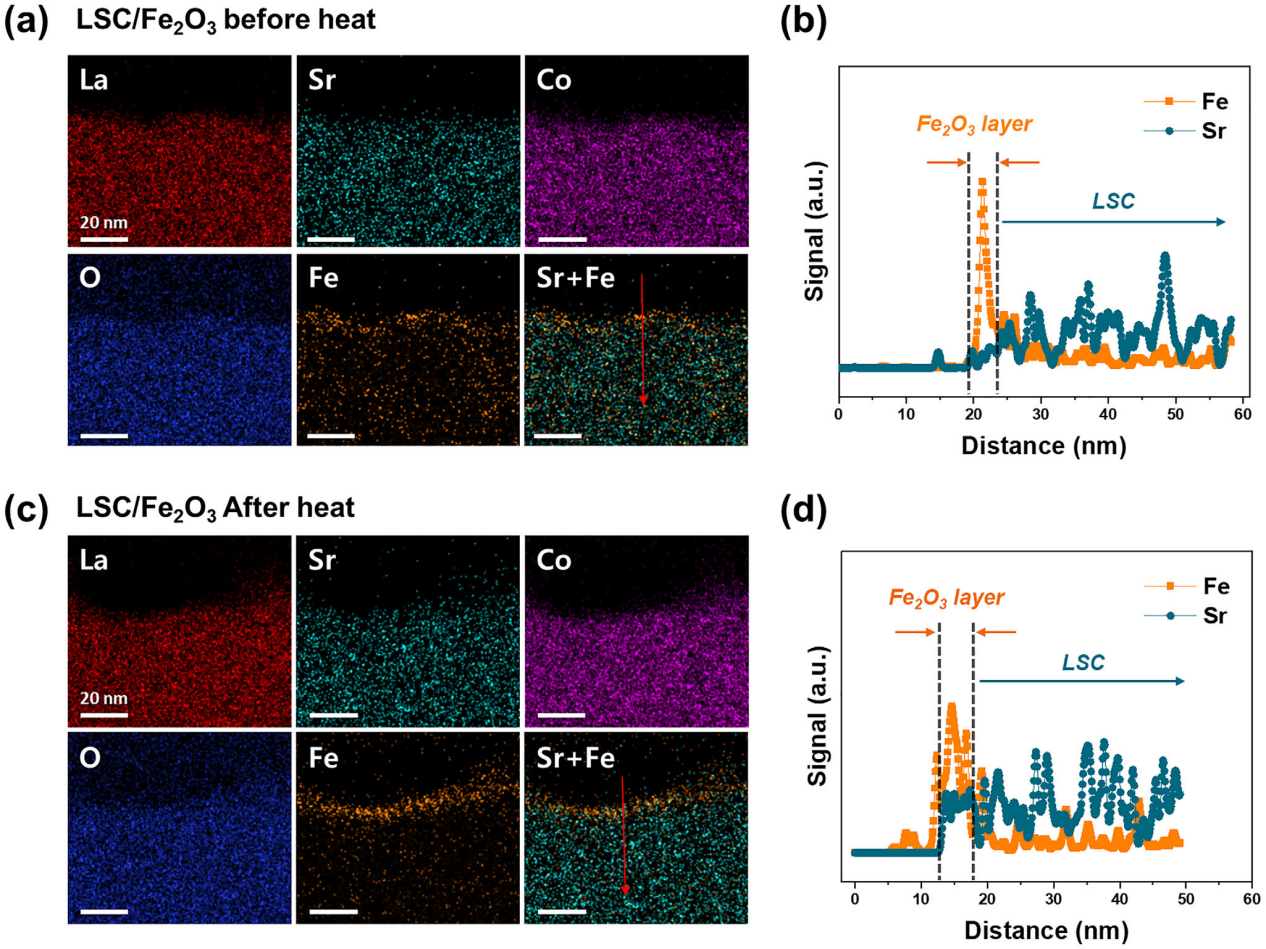
FIB‐TEM and corresponding EDS analysis on LSC/Fe_2_O_3_ samples (a,b) before and (c,d) after heat treatment at 600°C for 30 h. (a,c) show STEM‐EDS mapping and (b,d) present the line scan results throughout the Fe_2_O_3_ layer and LSC thin film.

FIB‐STEM and EDS analyses shown in Figure 6 further support the clear interaction between the LSC and Fe_2_O_3_ layer during heat treatment at 600°C. Figure 6a–d show the EDS elemental mapping results and corresponding Fe and Sr line‐scan profiles of LSC/Fe_2_O_3_ as‐dep and after heat treatment, respectively. Before heat treatment (Figure 6a,b), Sr was hardly detected in the Fe_2_O_3_ overlayer region. However, after annealing at 600°C for 30 h (Figure 6c,d), Sr was clearly observed inside the Fe_2_O_3_ layer. Taken together with the XRD results in Figure S10, these observations further indicate interfacial chemical interaction between the LSC thin film and the Fe_2_O_3_ overlayer, which could lead to the possible formation of Sr‐Fe‐O intermediate phases, such as Sr_2_Fe_2_O_5_. It should be noted, however, that the XRD data were obtained from a simulated environment using mixed SrO and Fe_2_O_3_ powders rather than the actual thin‐film configuration, and thus do not confirm the formation of a specific crystalline phase, e.g., Sr_2_Fe_2_O_5_. Nevertheless, the TEM‐EDS results clearly demonstrate that chemical interaction between LSC and Fe_2_O_3_ does take place in the real system, and this interfacial reaction plays a crucial role in enhancing the stability of the LSC/Fe_2_O_3_ sample during operation at 600°C (Figure 5a). Additional ECR analysis further shows that the performance enhancement at 600°C for the Fe_2_O_3_‐coated LSC sample originates not from the intrinsic property of the formed phase, such as Sr_2_Fe_2_O_5_, but rather from the chemical interaction between the LSC thin film and the Fe_2_O_3_ layer (Figure S11).

From these observations, we believe that the following indicators exist to determine the scavenger material and optimal coating conditions. First, the material to be coated must have a variety of intermediate phases with Sr species. Second, the amount of segregated Sr depends on the operating temperature, which should determine the appropriate coating thickness. Finally, since there is an activation energy that must be overcome for the reaction between Sr species and the coating material to take place, there exists a specific temperature window to vitalize the coating material as a scavenger. As such, we believe that these multiple factors have led to controversial and inconsistent results in the past studies, even when the same coating materials were employed.

Lastly, putting all these results together, we can answer the questions presented earlier. Through systematic investigation using a model thin‐film electrode, we observed that surface modification through oxide coating substantially improved the durability of LSC. By carefully controlling the thickness of the coating layer via ALD, we also found an optimal coating thickness, depending on the specific materials used, which varied with different coating thicknesses. Finally, using in‐depth surface chemical analysis, we revealed a new descriptor for selecting coating materials, that is, their reactivity with Sr species. Based on these findings, we can propose Fe_2_O_3_ as a new effective scavenger for improving durability and activity of oxygen exchange kinetics, as its applicability has also been confirmed in the actual porous electrode and electrochemical impedance spectroscopy (Figure S12). These insights open a clear path forward: the rational design of coating materials that fulfill these key criteria could unlock the next generation of highly durable and active oxygen electrodes.

## 4 Conclusions

In this study, we systematically investigated the effects of binary oxide coatings on the surface chemistry and oxygen exchange kinetics of La_0.6_Sr_0.4_CoO_3‐δ_ thin‐film electrodes. Using a model thin‐film platform combining PLD and ALD, we demonstrated that both HfO_2_ and Al_2_O_3_ coatings can effectively stabilize the oxygen exchange kinetics over time, although the underlying mechanisms differ. HfO_2_ improves stability by suppressing the formation of surface oxygen vacancies and minimizing the segregation of Sr ions toward the surface, while Al_2_O_3_ enhances durability through chemical reaction with segregated Sr species to form Sr‐Al‐O intermediate phases. The critical coating thickness for stabilization was found to differ significantly between the two materials, emphasizing the need to optimize coating conditions based on the chemical reactivity of the coating materials. Additional experiments using Fe_2_O_3_ as a new coating material further supported the importance of surface Sr reactivity and revealed a temperature window for effective scavenging. Taken together, these findings suggest that while the reactivity of coating materials with Sr species is a key design parameter, other factors (e.g., coating thickness, operating temperature, and activation energy for interfacial reactions) must also be considered in an integrated manner. This finding provides a comprehensive basis for the rational design of robust and durable oxygen electrodes for high‐temperature electrochemical energy systems.

## Author Contributions

WooChul Jung conceived and supervised the project. Jongsu Seo and WooChul Jung contributed to conceptualization. Jongsu Seo and SungHyun Jeon performed the following experiments: electrochemical analysis and physical/chemical properties such as SEM, TEM, ToF‐SIMS, XPS, and EIS analyses. DongHwan Oh conducted FIB‐TEM analysis. San Kwak, Hyunseung Kim, and Jinwook Kim contributed to thin‐film sample fabrication and optimization. Bonjae Koo contributed to the elucidation of XPS results. Jongsu Seo, SungHyun Jeon, and Jeong Hwan Kim performed surface coating of oxide layer using atomic layer deposition. Jongsu Seo and SungHyun Jeon contributed to the writing of the manuscript. WooChul Jung contributed to supervising the manuscript.

## Conflicts of Interest

The authors declare no conflicts of interest.

## Supporting information




**Supporting File**: adma72205‐sup‐0001‐SuppMat.docx.

## Data Availability

The data that support the findings of this study are available from the corresponding author upon reasonable request.
