# Supplementary material for: Effect of Atomic Layer Deposition of Ultra‐Thin Oxide on Reactivity and Durability of Perovskite Oxygen Electrodes
Source: Adv Mater. 2026 Jan 19;38(32):e13655. doi: 10.1002/adma.202513655 (PMC13244811; doi:10.1002/adma.202513655)
Supplement: Supplementary file 1 — Supporting File: adma72205‐sup‐0001‐SuppMat.docx. [file ADMA-38-e13655-s001.docx]

Effect of Atomic Layer Deposition of Ultra-thin Oxide on Reactivity and Durability of Perovskite Oxygen Electrodes

Jongsu Seo^a+^, SungHyun Jeon^b,c+^, Hyunseung Kim^b^, San Kwak^d^, DongHwan Oh^e^ , Bonjae Koo^f^, Jinwook Kim^c^, Jeong Hwan Kim^g^, and WooChul Jung^b,d*^

^a^ Hydrogen Research Department, Korea Institute of Energy Research (KIER), Daejeon, 34129 Republic of Korea

^b^ Research Institute of Advanced Materials, Seoul National University, Seoul, 08826 Republic of Korea

^c^ Department of Materials Science and Engineering, Northwestern University, Evanston, IL, 60208 USA

^d^ Department of Materials Science and Engineering, Seoul National University, Seoul, 08826 Republic of Korea

^e^ Korea Advanced Institute of Science and Technology (KAIST), Daejeon, 34141 Republic of Korea

^f^ School of Chemistry and Energy, Sungshin Women’s University, Seoul, 02844 Republic of Korea

^g^ Department of Materials Science and Engineering, Hanbat National University, Daejeon, 34158 Republic of Korea

+ These authors contributed equally to this work.

*Corresponding author

E-mail address: [wcjung@snu.ac.kr](mailto:wcjung@snu.ac.kr) (W. Jung)


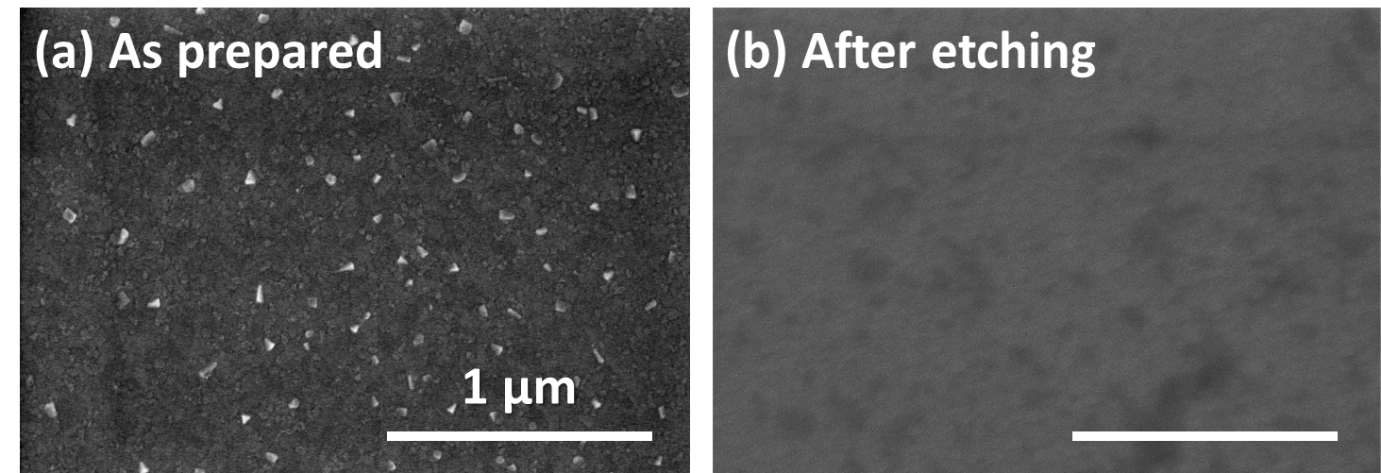


**Figure S1**. SEM images of LSC thin film: (a) as-prepared and (b) after etching.


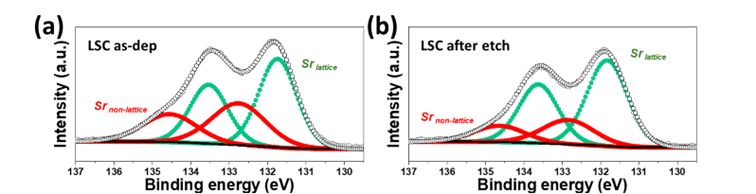


**Figure S2.** XPS Sr 3d spectra of LSC thin film (a) as-deposited and (b) after chemical etching.


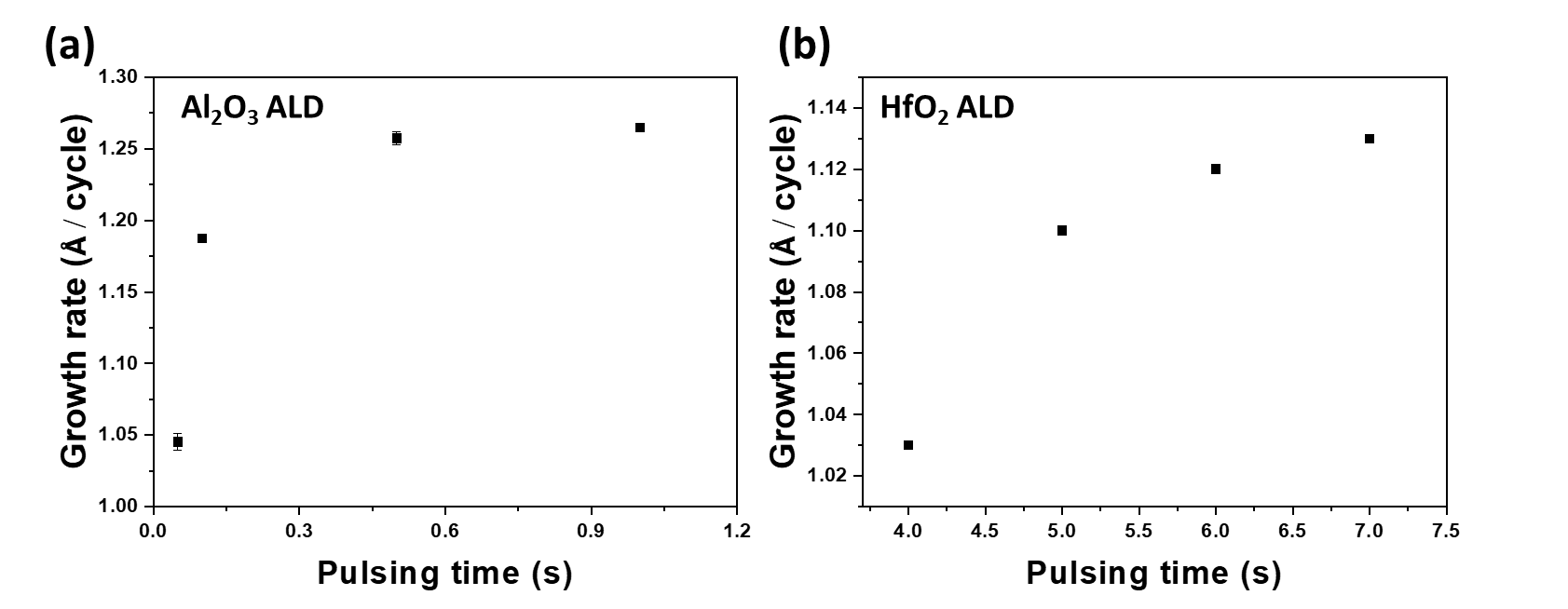


**Figure S3**. Growth rate in precursor pulsing time for (a) Al_2_O_3_ and (b) HfO_2_ ALD coating layer


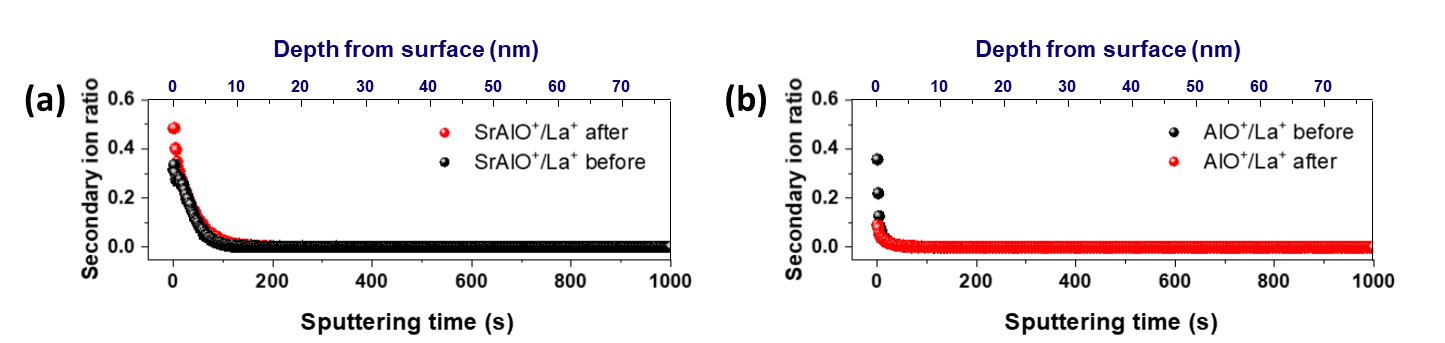


**Figure S4**. ToF-SIMS analysis of Al_2_O_3_-coated LSC thin film: Positive secondary ion signal ratio of (a) SrAlO^+^/La^+^ and (b) AlO^+^/La^+^ before and after heat treatment.


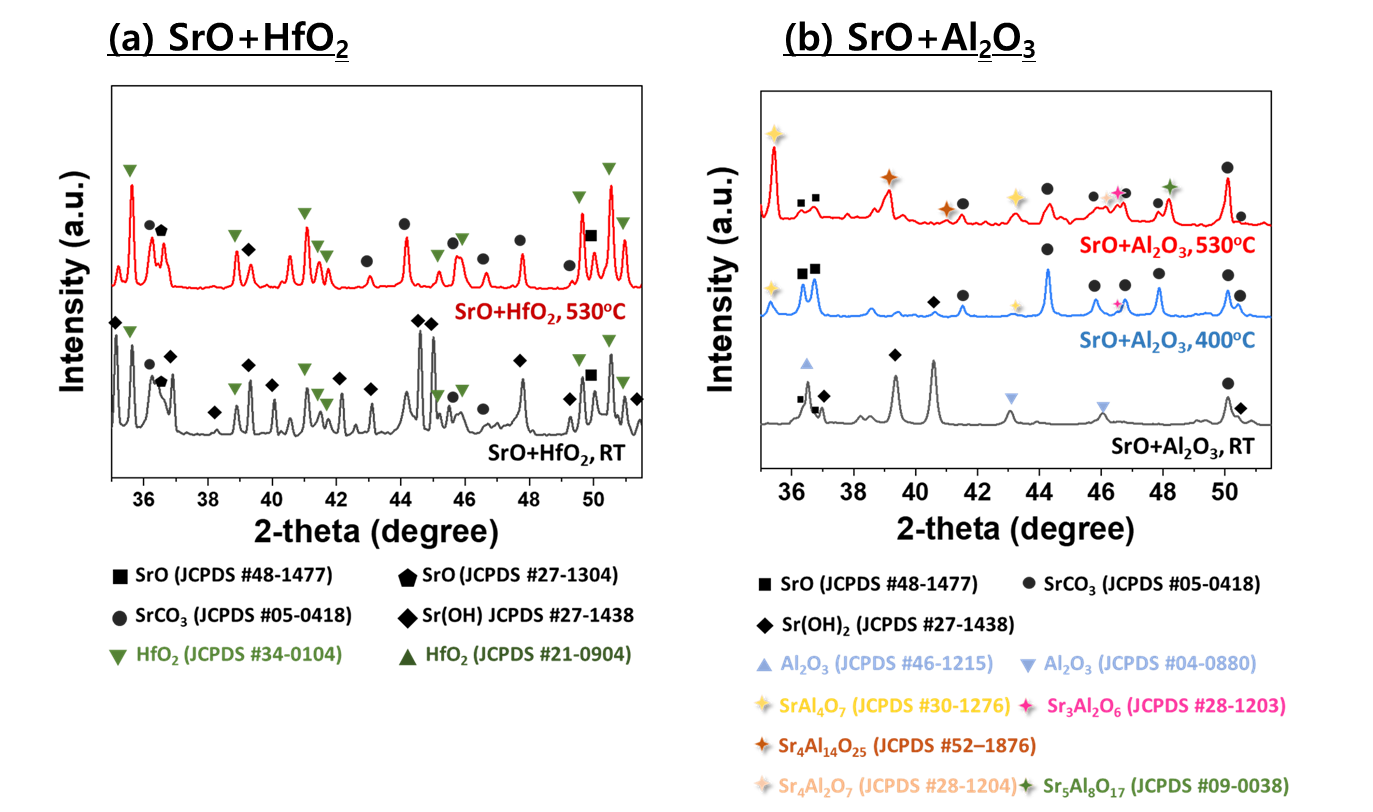


**Figure S5**. XRD analysis of SrO powder mixed with (a) HfO_2_, (b) Al_2_O_3_, and after heat treatment in different temperatures.

XRD analyses was performed on mixed oxide powders at various annealing temperatures, in addition to the original results. To better simulate realistic conditions, the initial state of SrO powder was prepared to contain coexisting phases such as Sr(OH)_2_ and SrCO_3_, which are typically present under ambient exposure.

As shown in **Figure S6(a)**, the SrO + HfO_2_ sample retained the same phases up to 530°C as those observed at room temperature. The only difference was a gradual decrease in the Sr(OH)_2_ phase accompanied by an increase in SrO and SrCO_3_ peaks upon heating.

In contrast, **Figure S6(b)** shows that in the SrO + Al_2_O_3_ mixture, no reaction occurred at room temperature, where only SrO, SrCO_3_, Sr(OH)_2_, and Al_2_O_3_ peaks were detected. However, upon heating to 400°C, the Sr(OH)_2_ and SrO peaks began to decrease, while new peaks corresponding to intermediate phases such as SrAl_4_O_7_ and Sr_3_Al_2_O_6_ emerged. At 530°C, these intermediate phase peaks became more pronounced, accompanied by the formation of additional compounds such as Sr_4_Al_14_O_25_ and Sr_4_Al_2_O_7_, with further consumption of SrO and SrCO_3_.

These results suggest that Al_2_O_3_ can effectively act as a scavenging layer, reacting with Sr-containing species even at relatively low temperatures (~400°C).


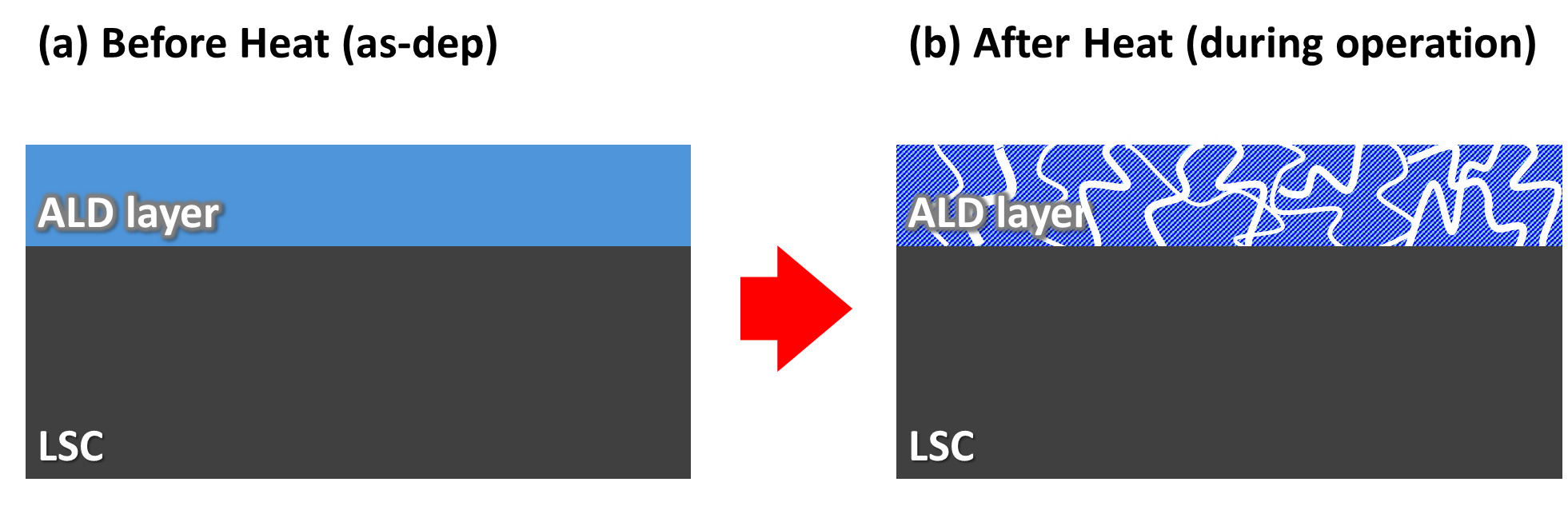


**Figure S6**. Schematic illustration of changes in ALD coating layer during heat treatment: (a) as-dep and (b) after heat

**Table S1.** BET analysis on LSC and LSC/Al_2_O_3_ powder.

|  | **Specific surface area (m^2^/g)** | **Adsorption volume of monolayer (cm^3^/g)** |
| --- | --- | --- |
| **LSC bare** | **4.478** | **1.029** |
| **LSC/Al_2_O_3_ as-dep** | **4.699** | **1.080** |
| **LSC/Al_2_O_3_ after heat** | **5.282** | **1.214** |
| **Surface exposure during heat**  **(BET _after heat_ / BET _as-dep_ - 1)** | **12.4%** | |

**Figure S7** illustrates a schematic of the expected evolution of the ALD coating layer after annealing. The as-deposited ALD layer is amorphous and dense, whereas after heat treatment, it is expected to become more crystalline, forming micro- or nanopores within the film and thereby increasing the exposed surface area of the underlying LSC.

To support this, we performed BET analyses for LSC/Al_2_O_3_ powder samples before and after heat treatment. **Table S1** summarizes the BET results of LSC and LSC/Al_2_O_3_ powders before and after annealing. Notably, the specific surface area of LSC/Al_2_O_3_ increased by approximately 12% after heat treatment. This increase is likely due to the partial porosity development of the Al_2_O_3_ layer, which allows more N_2_ adsorption sites to become accessible, as illustrated in **Figure S7**.

Furthermore, several studies have reported how ALD-derived oxide coatings evolved at elevated temperatures, showing trends consistent with our findings. Furthermore, several studies have reported how ALD-derived oxide coatings evolved at elevated temperatures. George *et al*. demonstrated that the high temperature annealing induced structural transformation of the Al_2_O_3_ ALD layer, resulting in pore formation and an increased BET surface area.^[1]^ HR-TEM observation by Gong *et al*. directly revealed that even a relatively thick (~20 nm) ALD coating layer undergoes crystallization and crack formation after high-temperature annealing, leading to the formation of micro- or nanopores.^[2]^ Seo et al. showed through ECR analysis that the exposed surface area of an ~1.5 nm Al_2_O_3_ layer increased by around 15% after heat treatment.^[3]^ These findings are consistent with our observation of a ~12% increase, suggesting that the coverage of the ALD layer can decrease, *i.e.,* the exposed LSC surface can increase, by approximately 12~15% at elevated temperature.


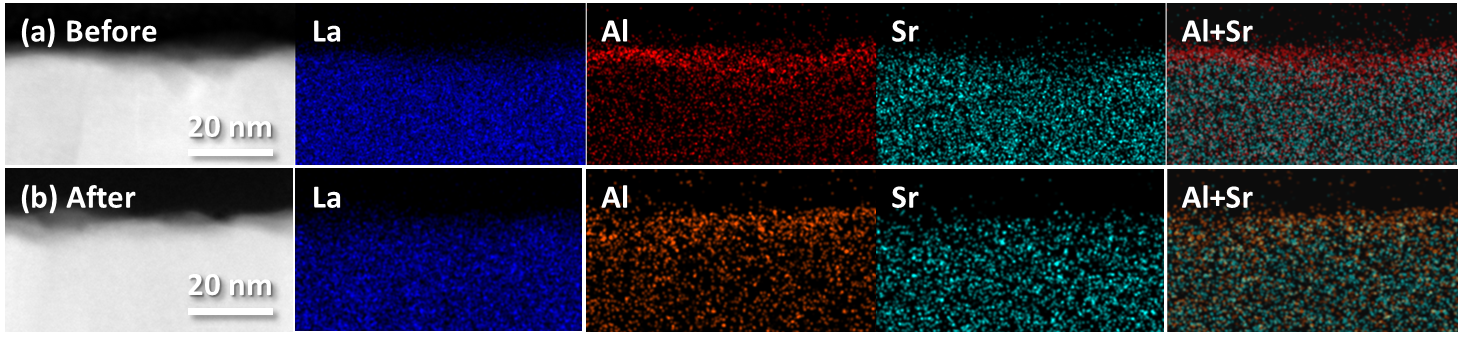


**Figure S7**. Additional HAADF-STEM and EDS results (a) before and (b) after heat treatment.


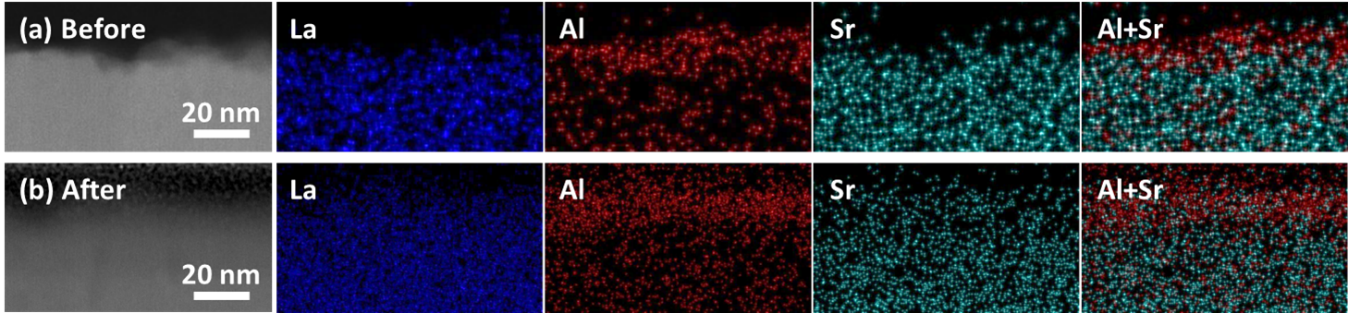


**Figure S8**. Additional HAADF-STEM and EDS results (a) before and (b) after heat treatment.


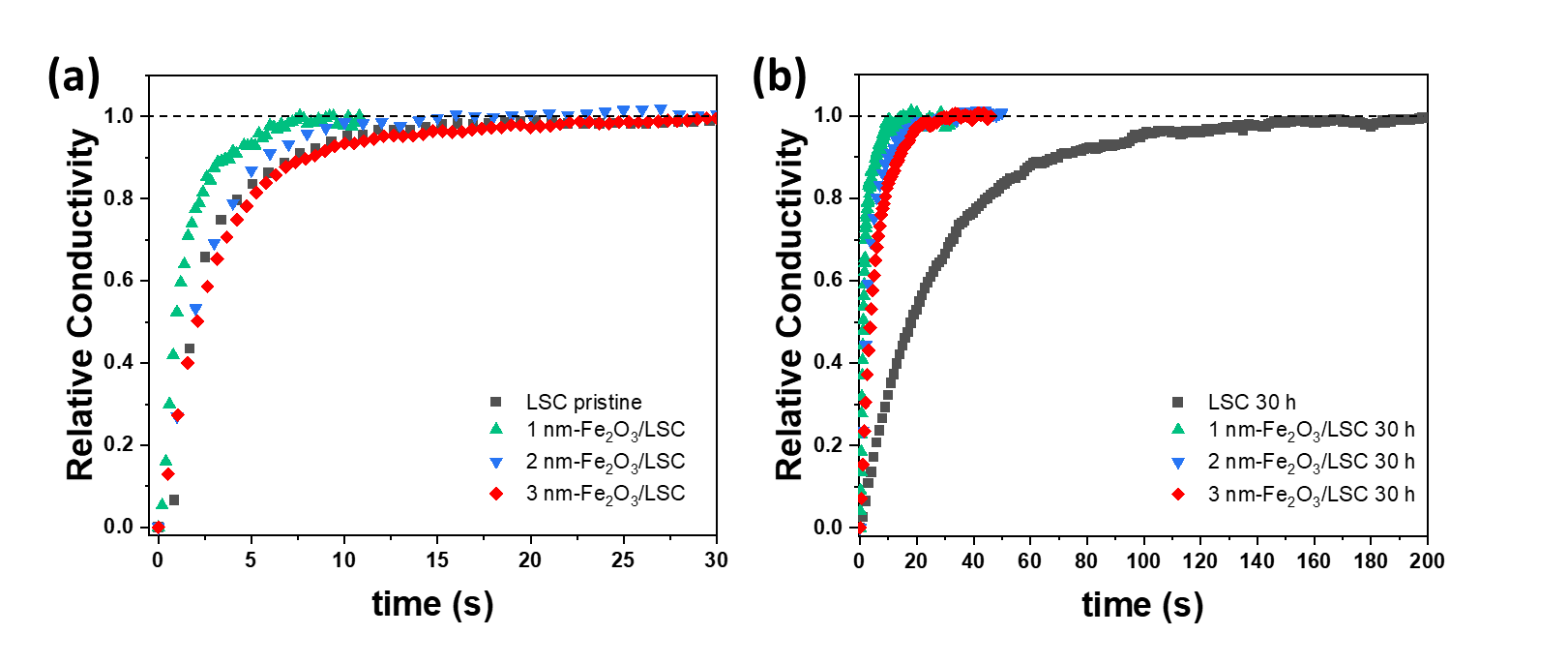


**Figure S9**. Electrical conductivity relaxation curve of LSC and Fe_2_O_3_-coated LSC thin film (a) initial and (b) after 30 h of operation at 600°C.


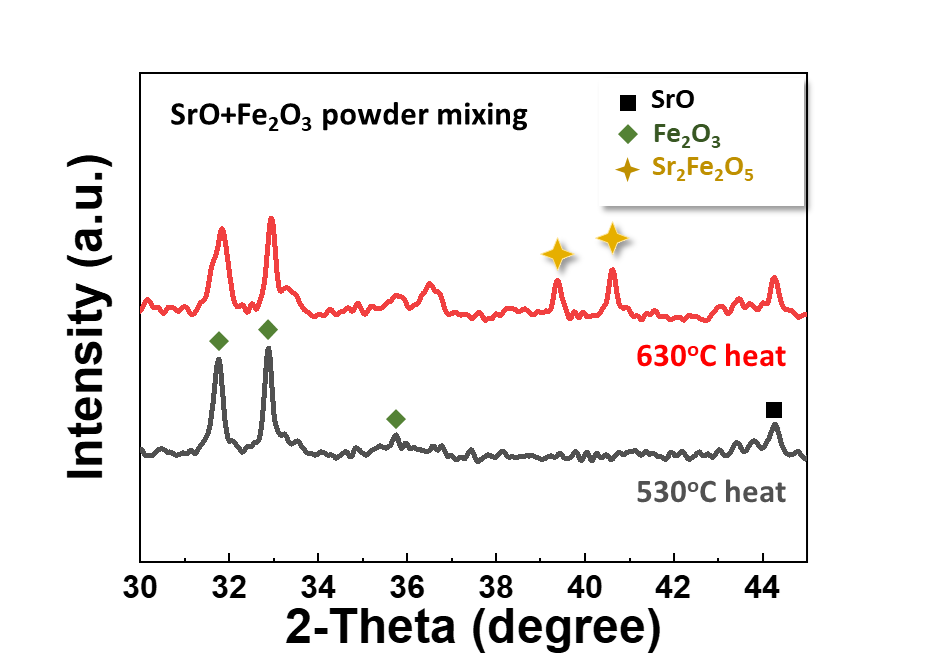


**Figure S10**. XRD analysis of mixed powder of SrO andFe_2_O_3_ after different heat treatment temperature of 530°C and 630°C for 2 h.


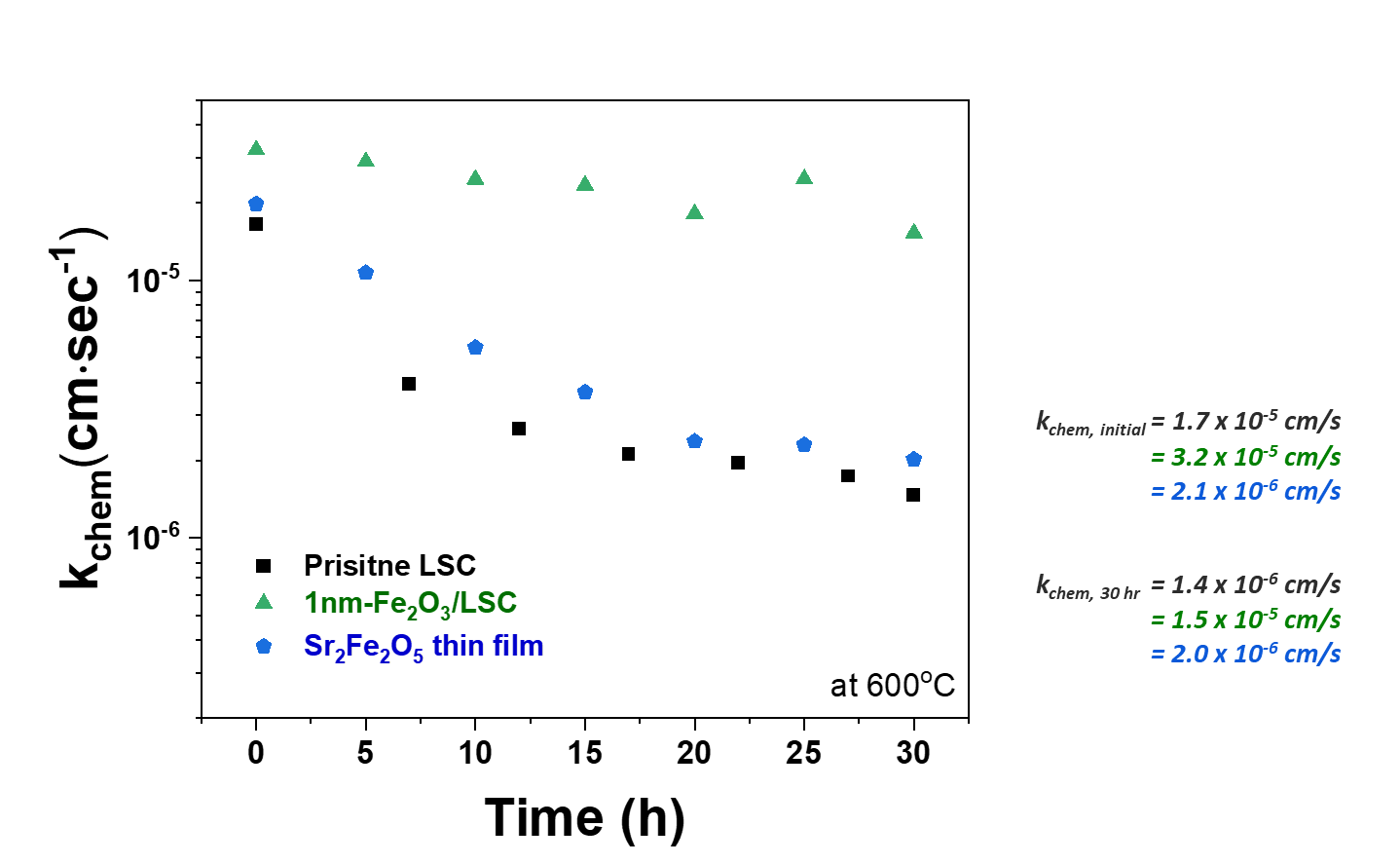


**Figure S11.** ECR analysis on LSC, LSC/Fe_2_O_3_, and Sr_2_Fe_2_O_5_ thin film: A comparison in a stability test during 30 hours at 600°C.


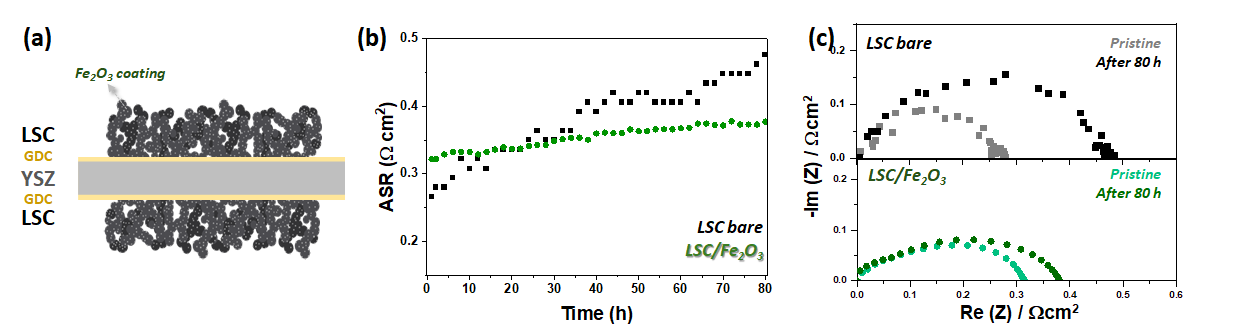


**Figure S12.** Short-term stability in electrochemical impedance spectroscopy of porous electrode comparing LSC bare and Fe_2_O_3_-infiltrated LSC half-cell at 650°C: (a) Schematic configuration of porous half-cell, (b) short-term stability results, and (c) Nyquist plot of pristine and after 80 h samples.

To mimic the actual electrode structure, we also fabricated SOFC half-cell with porous La_0.6_Sr_0.4_CoO_3-d_ (LSC) electrodes. LSC ink slurry was prepared by mixing an ink vehicle and LSC powder in a 1:1 weight ratio along with a small amount of ethanol, and the resulting ink was screen-printed onto the both sides of 8 mol%-Y_2_O_3_-stabilized ZrO_2_ (YSZ) electrolytes with PLD-deposited Gd_0.1_Ce_0.9_O_2-d_ buffer layers. The half-cells were then sintered at 950°C for 1 hour in air. The Fe_2_O_3_ was incorporated on both sides of porous LSC electrodes. To better reflect practical electrode fabrication route, the infiltration process was selected for Fe_2_O_3_ coating rather than thin-film deposition. A 10 mM Fe(NO_3_)_3_∙9H_2_O solution (10 μL per electrode side) was infiltrated, followed by calcination at 650°C.

The stability test in electrochemical impedance spectroscopy (EIS) for pristine LSC and Fe_2_O_3_-coated LSC porous half-cell were conducted to determine whether the Fe_2_O_3_ coating is effective in actual porous electrode configuration. The EIS measurement was carried out over a frequency range of 1 MHz to 10 mHz with an AC perturbation amplitude of 40 mV.

**Reference in Supporting Information**

[1] C. George, P. Littlewood, P. C. Stair, *ACS Appl Mater Interfaces* 2020, *12*, 20331.

[2] Y. Gong, R. L. Patel, X. Liang, D. Palacio, X. Song, J. B. Goodenough, K. Huang, *Chemistry of Materials* 2013, *25*, 4224.

[3] J. Seo, N. Tsvetkov, S. J. Jeong, Y. Yoo, S. Ji, J. H. Kim, J. K. Kang, W. C. Jung, *ACS Appl Mater Interfaces* 2020, *12*, 4405.
